# Supplementary material for: Dynamic Distribution of Gut Microbiota in Pigs at Different Growth Stages: Composition and Contribution
Source: Microbiol Spectr. 2022 May 18;10(3):e00688-21. doi: 10.1128/spectrum.00688-21 (PMC9241710; doi:10.1128/spectrum.00688-21)
Supplement: SUPPLEMENTAL FILE 1 — Supplemental material. Download spectrum.00688-21-s001.pdf, PDF file, 1.9 MB [file spectrum.00688-21-s001.pdf]

Supplementary Material and Methods

Dynamic distribution of gut microbiota in pigs at different growth stages: composition and contribution

Yuheng Luo[1, †], Wen Ren[1, 2, †], Hauke Smidt[3], André-Denis G. Wright[4], Bing Yu[1], Ghislain Schyns[5], Ursula M. McCormack[6], Aaron J. Cowieson[5], Jie Yu[1], Jun He[1], Hui Yan[1], Jinlong Wu[2], Roderick I. Mackie[7], and Daiwen Chen[1, *]

[1] Key Laboratory for Animal Disease-Resistance Nutrition of Ministry of Education of China, Key Laboratory for Animal Disease-Resistance Nutrition and Feed of Ministry of Agriculture of China, Key laboratory of Animal Disease-resistant Nutrition of Sichuan Province, and Animal Nutrition Institute, Sichuan Agricultural University, Chengdu, China, Chengdu 611130, People's Republic of China.

[2] DSM (China) Animal Nutrition Research Center Co., Ltd, Bazhou 065799, People's Republic of China.

[3] Laboratory of Microbiology, Wageningen University, Wageningen, Netherlands

[4] Department of Animal Sciences, College of Agricultural, Human, and Natural Resource Sciences, Washington State University, Pullman, WA 99163

[5] DSM Nutritional Products Ltd, Kaiseraugst, Switzerland

[6] DSM Nutritional Products France, Centre De Recherche En Nutrition Animale, Saint Louis, France

[7] Department of Animal Sciences, and Carle R. Woese Institute for Genomic Biology, University of Illinois, Urbana, IL 61801, USA

[†] These authors contributed equally to this study.

[*] Corresponding authors: chendwz@sicau.edu.cn (D. Chen)

Supplement figures (Figure S1-S10):

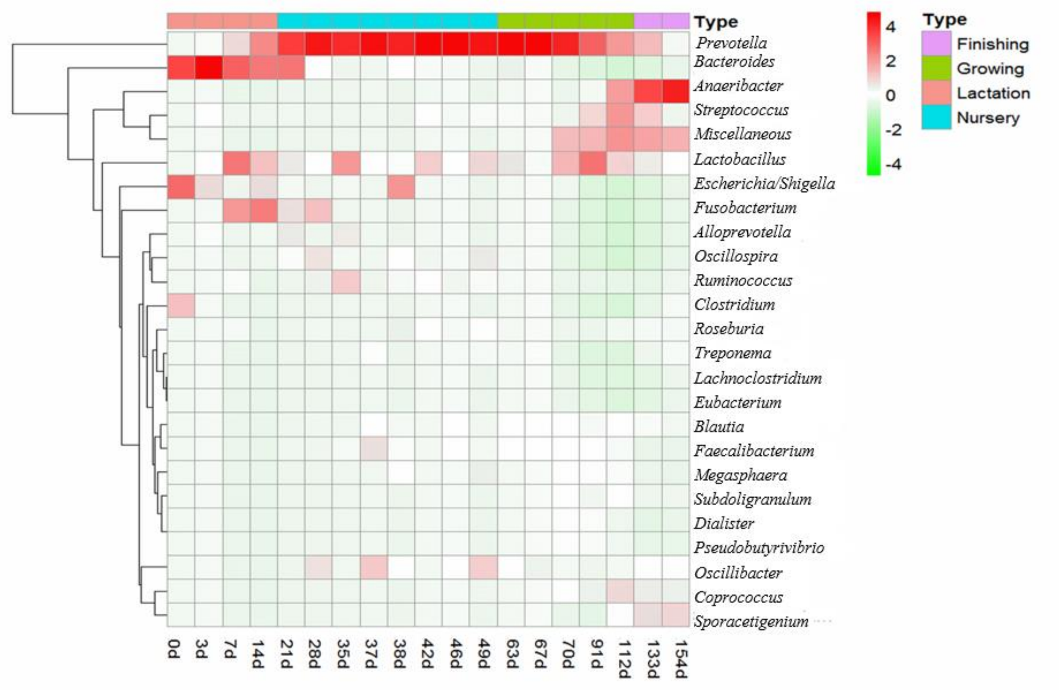

Figure S1. The dynamic distribution of bacteria at genus level in the gut of pigs at different ages. The relative abundance of top 25 genera are shown. Data are summarized from 63 publications.

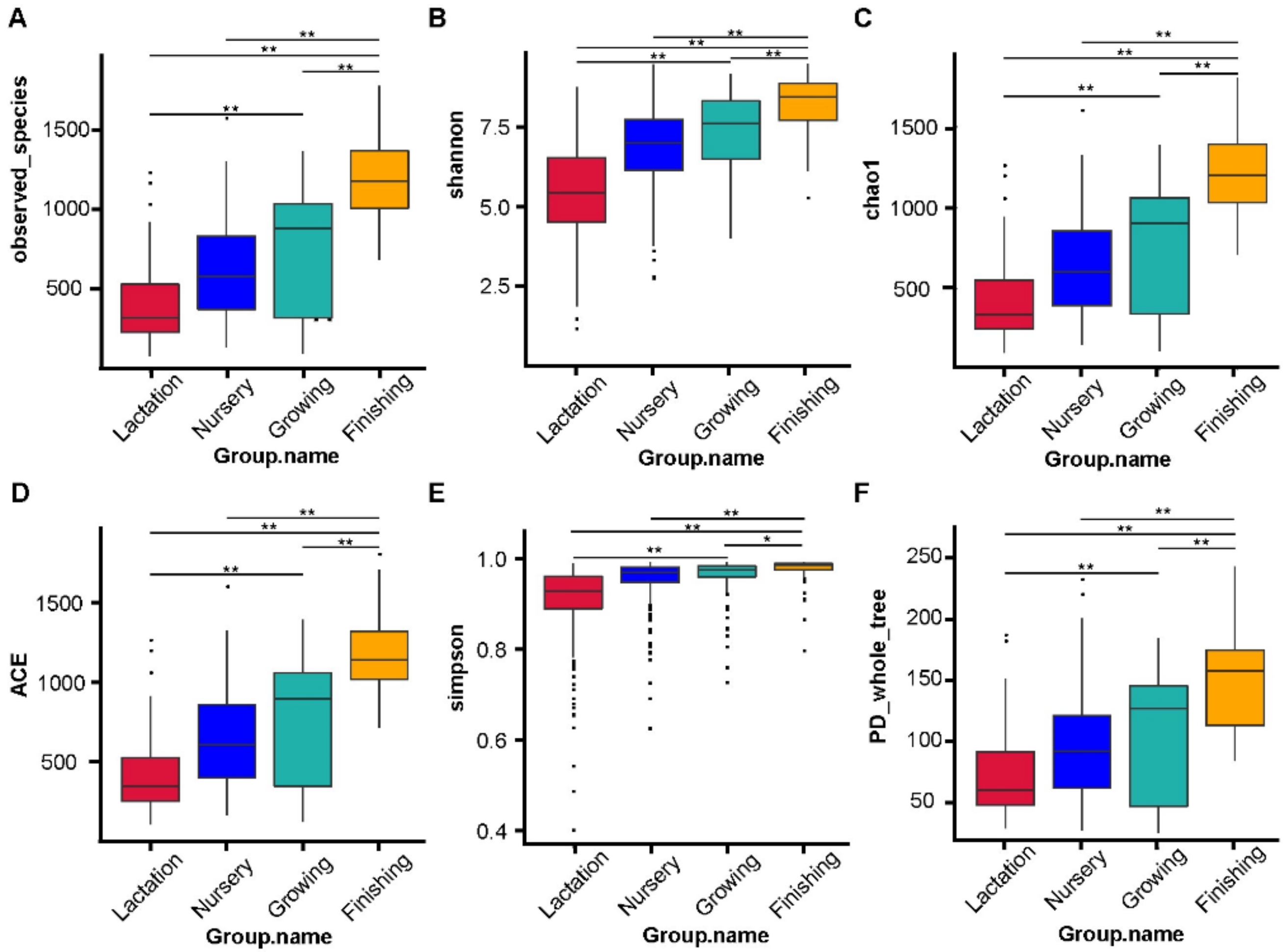

Figure S2. Changes of richness and diversity of microbial community in the gut of pigs at different growth stages. A, observed species; B, Shannon; C, chao1;D, ACE; E, Simpson; F, PD whole tree. **, $P < 0.01$ (Tukey's test). Stages lactation, nursery, growing and finishing are distinguished in red, blue, green and orange, respectively

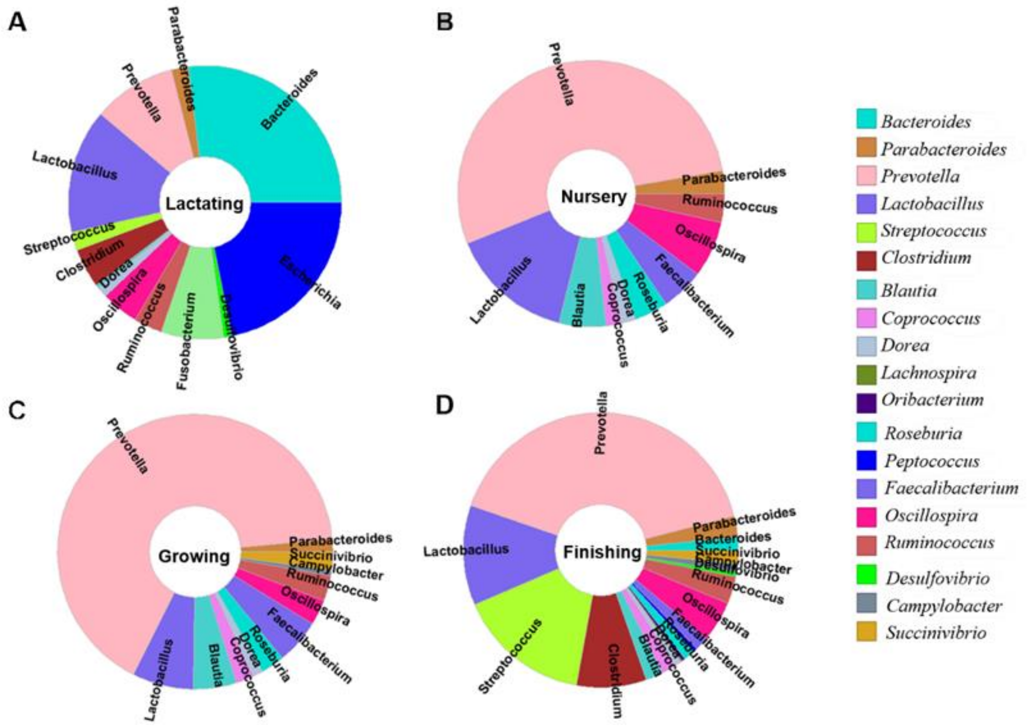

Figure S3. The pie charts of the 19 core bacterial genera and their contribution in each growth phase. A, lactating piglets; B, nursery piglets; C, growing pigs; D, finishing pigs.

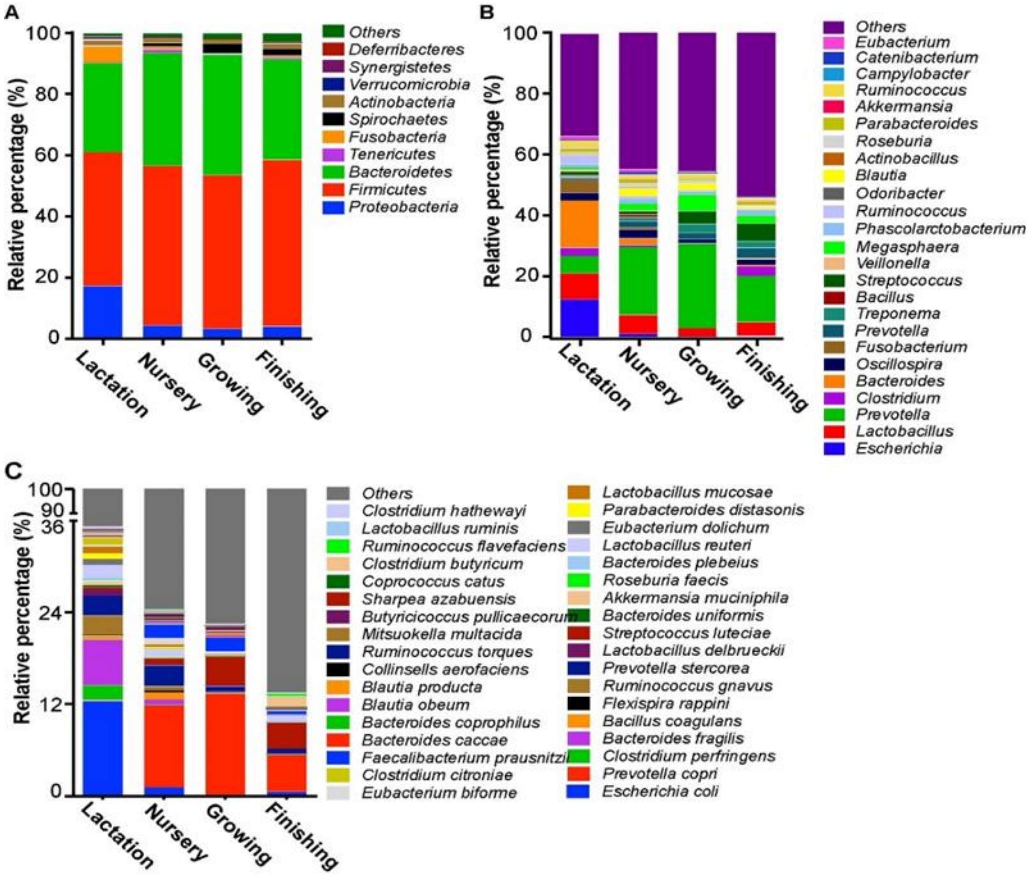

Figure S4. The most abundant bacteria at phylum, genus and species levels in the gut of pigs at different growth phases. A, the top 10 bacterial phyla; B, the top 25 genera; C, the top 35 species.

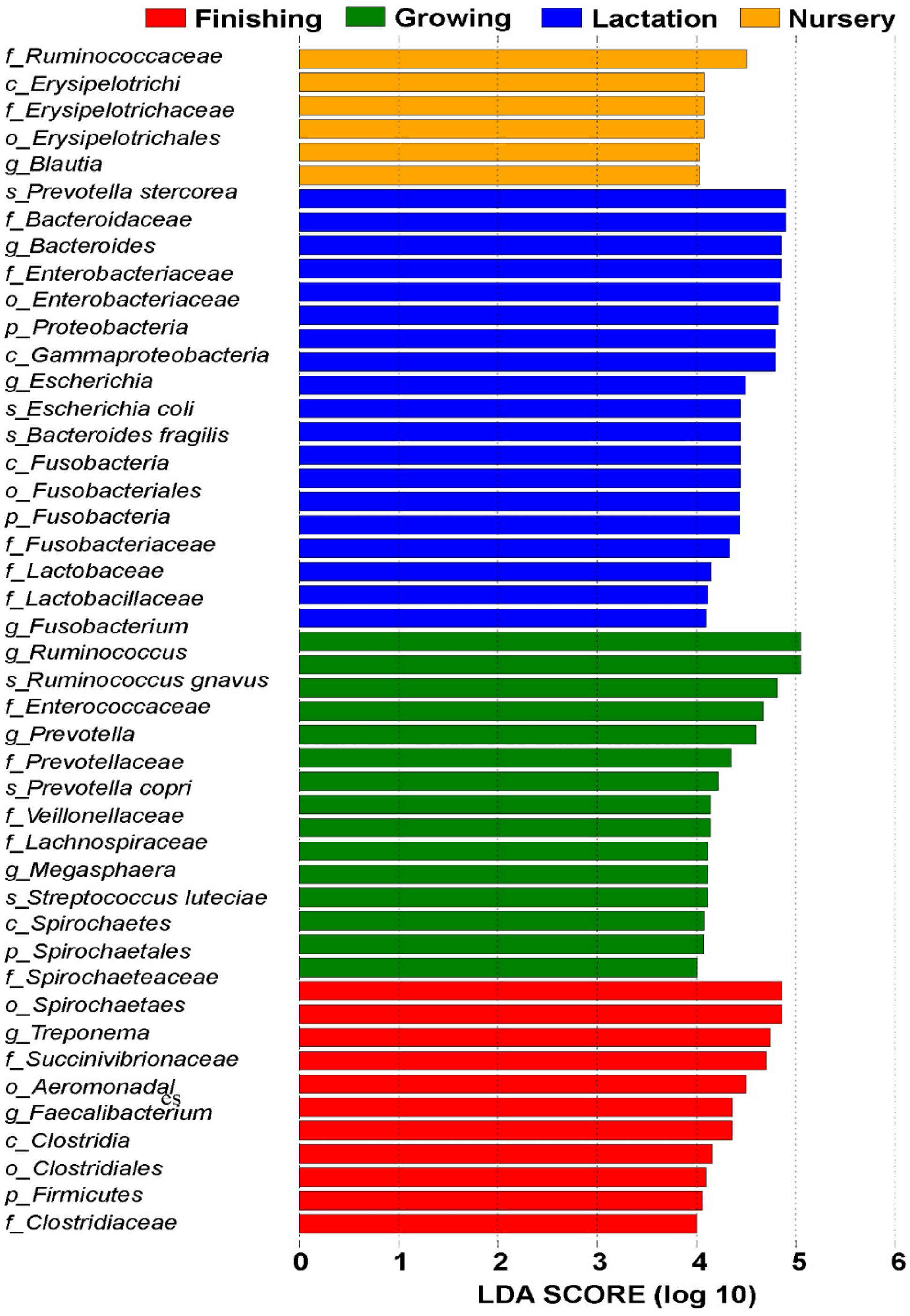

Figure S5. Differentially abundant bacteria at all taxonomical levels in the gut of pigs at each growth stage assessed using a linear discriminant analysis (LDA) with effect size (LEfSe) measurement. Only those genera with an LDA score ($\log_{10}$) > 4.0 are displayed. The 4 growth stages are differentiated by colors: blue for lactating piglets, brown for nursery piglets, green for growing pigs, and red for finishing pigs.

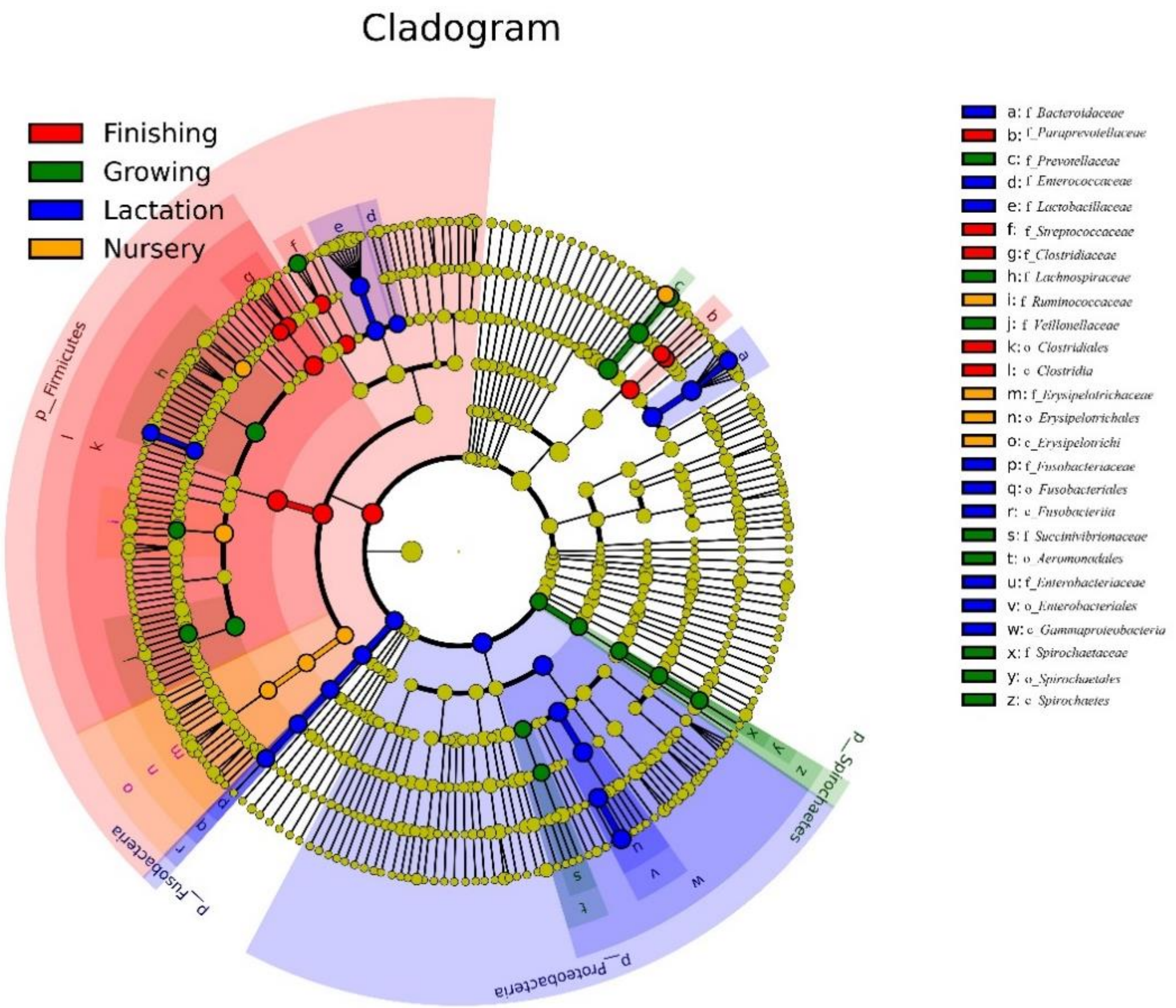

Figure S6. The enriched bacteria at different levels in the gut of pigs at different growth stages. p_, phylum; c_, class; o_, order; f_, family.

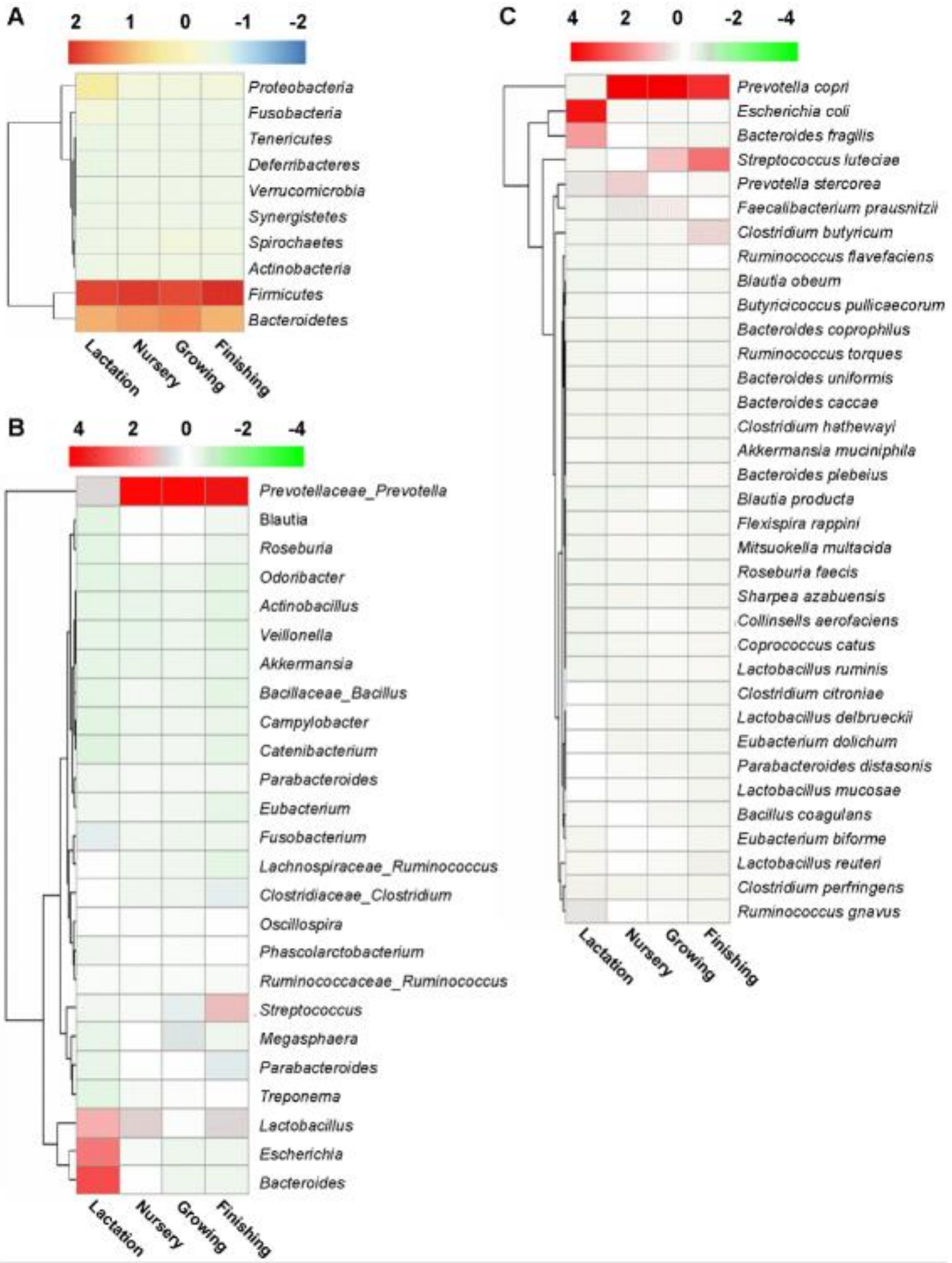

Figure S7. Heatmap of the most abundant bacteria at the phylum, genus and species levels in the gut of pigs at different growth phases. A, the 10 most abundant phyla; B, the 25 most abundant genera; C, the 35 most abundant species.

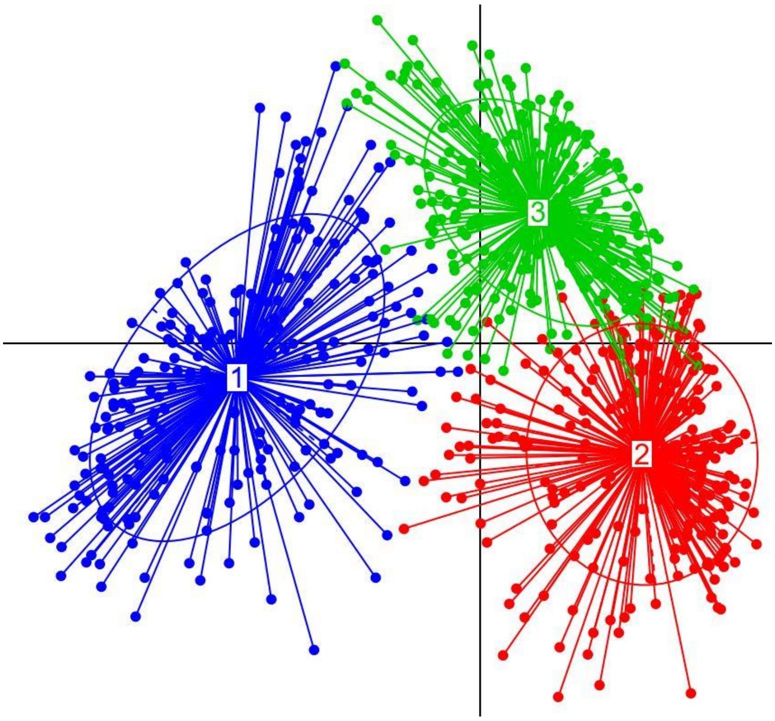

Figure S8. The distribution of 3 microbial enterotypes in the gut of pigs throughout the whole growth phase. 1, *Bacteroides*-*Escherichia*-*Lactobacillus*; 2, *Prevotella*-*Lactobacillus*-*Megasphaera*; 3, *Prevotella*-*Lactobacillus*-*Treponema*.

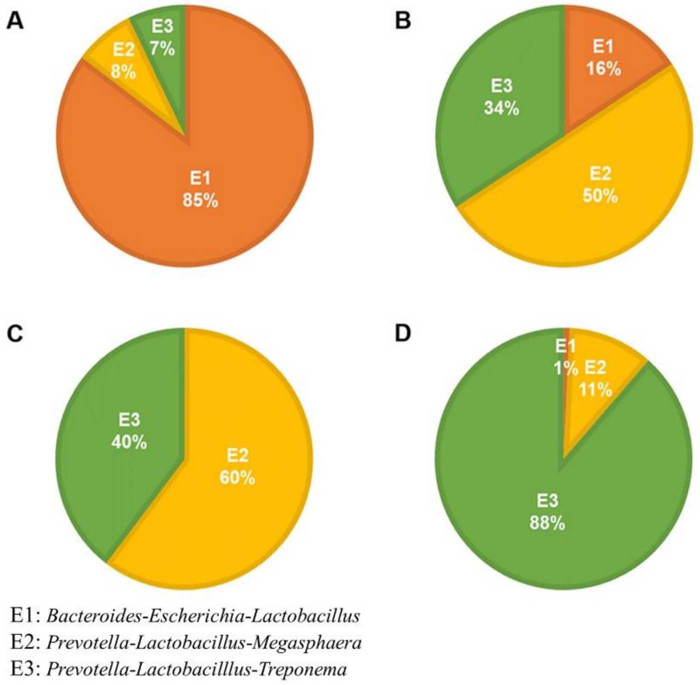

Figure S9. The contribution of different enterotypes in the gut of pigs at each growth phase. A, lactating piglets; B, nursery piglets; C, growing pigs; D, finishing pigs. Enterotypes are differentiated by colors: orange for E1 (*Bacteroides*-*Escherichia*-*Lactobacillus*), yellow for E2 (*Prevotella*-*Lactobacillus*-*Megasphaera*), and green for E3 (*Prevotella*-*Lactobacillus*-*Treponema*).

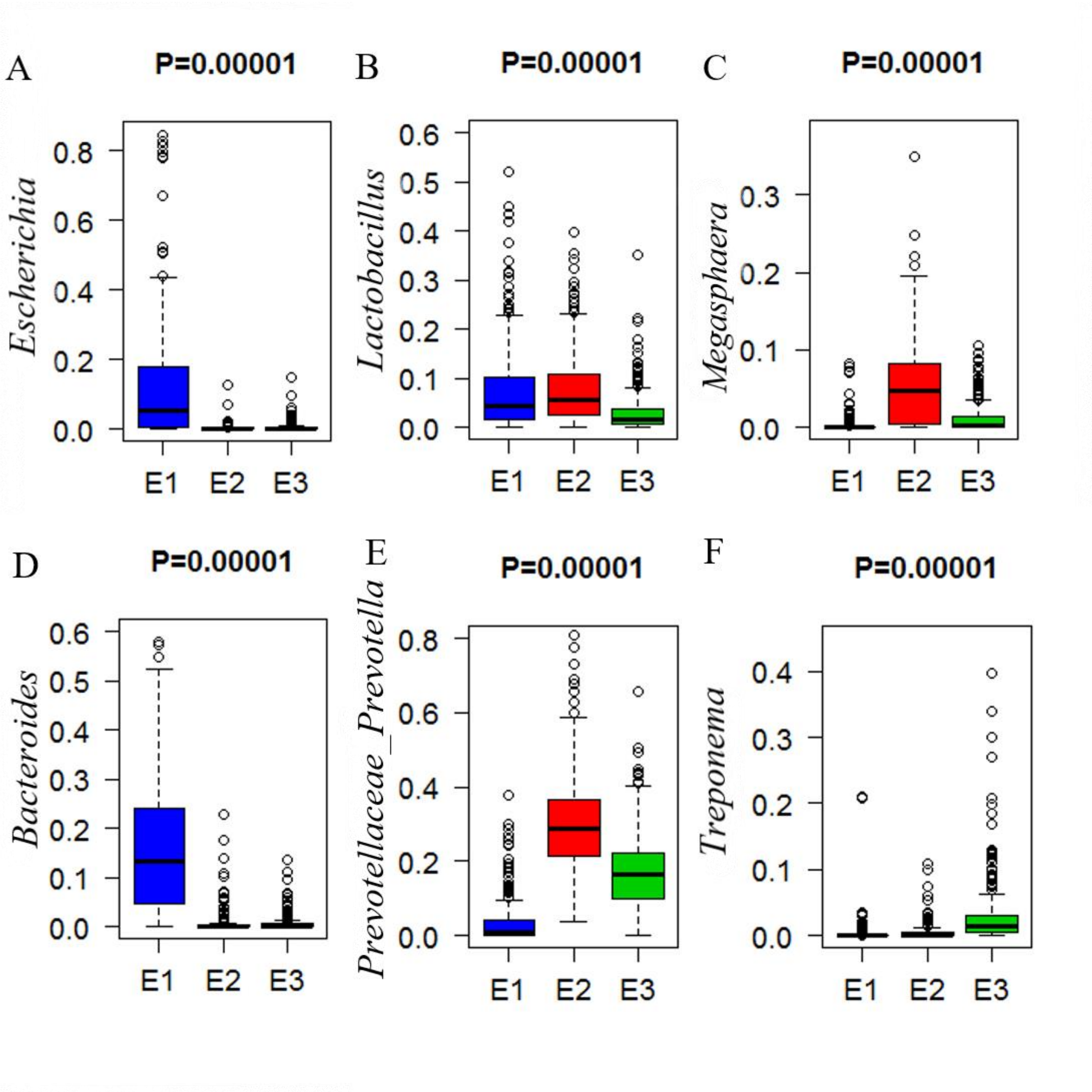

Figure S10. The relative abundance (%) of the 6 dominate genera contributing to the 3 enterotypes. A, *Escherichia*; B, *Lactobacillus*; C, *Megasphaera*; D, *Bacterioides*; E, *Prevotelllaceae_Prevotella*; F, *Treponema*. Enterotypes are differentiated by colors: blue for E1 (*Bacteroides*-*Escherichia*-*Lactobacillus*), red for E2 (*Prevotella*-*Lactobacillus*-*Megasphaera*) and green for E3 (*Prevotella*-*Lactobacillus*-*Treponema*).

Supplement tables (Table S1-S7)

Table S1. The three most abundant genera in the gut of suckling piglets (lactation period)

Age	Three most abundant genera	Reference
0 day	*Bacteroides* (29.60%), *Escherichia* (24.60%), and *Clostridium* (12.30%)	(1)
7 day	*Bacteroides* (27.84%), *Lactobacillus* (24.50%) and *Fusobacterium* (19.90%)	(1-3)
14 day	*Bacteroides* (16.42%), *Fusobacterium* (16.10%) and *Prevotella* (14.76%)	(1, 2, 4)
21 day	*Prevotella* (17.93%), *Bacteroides* (13.31%) and *Fusobacterium* (4.97%)	(1, 2, 4-6)

Table S2. The three most abundant genera in the gut of nursery, growing and finishing pigs

Age	Three most abundant genera	Reference
28 day	*Prevotella* (28.55%), *Fusobacterium* (10.00%), and *Oscillibacter* (6.53%)	(1, 3, 5, 7-9)
35 day	*Prevotella* (25.72%), *Lactobacillus* (14.04%), and *Ruminococcus* (8.60%)	(1, 2)
37 day	*Prevotella* (23.00%), *Oscillibacter* (7.00%), and *Faecalibacterium* (5.00%)	(10)
38 day	*Prevotella* (24.05%), *Escherichia* (13.12%), and *Roseburia* (4.42%)	(8)
42 day	*Prevotella* (24.60%), *Lactobacillus* (6.6%), and *Oscillospira* (2.80%)	(3, 4, 9, 11, 12)
46 day	*Prevotella* (38.00%), *Ruminococcus* (4.00%), and *Lactobacillus* (3.00%)	(10, 12)
49 day	*Prevotella* (20.05%), *Lactobacillus* (5.38%), and *Oscillibacter* (6.03%)	(8, 13)
63 day	*Prevotella* (26.98%), *Lactobacillus* (4.74%), and *Bacteroides* (3.60%)	(10, 14)
67 day	*Prevotella* (27.00%), *Oscillibacter* (3.00%), and *Faecalibacterium* (1.00%)	(10)
70 day	*Prevotella* (26.06%), *Lactobacillus* (10.98%), and *Miscellaneous* (10.50%)	(10, 15, 16)
84 day	*Prevotella* (19.00%), *Oscillibacter* (3.00%), and *Treponema* (1.70%)	(10)
91 day	*Prevotella* (15.90%), *Lactobacillus* (14.50%), and *Miscellaneous* (9.23%)	(15, 16)
112 day	*Miscellaneous* (9.67%), *Streptoccus* (9.51%), and *Prevotella* (9.38%)	(15, 16)
133 day	*Anaeribacter* (16.44%), *Miscellaneous* (9.77%), and *Prevotella* (8.01%)	(15, 16)
154 day	*Anaeribacter* (24.29%), *Miscellaneous* (10.53%), and *Sporacetigenium* (7.11%)	(15, 16)

Table S3. Details of studies included in the meta-analysis

Treatment	Sample choose	Creep feed	No. of samples: Lactation (0-20 d)	Nursery (21-60 d)	Growing (61-116 d)	Finishing (117-174 d)	Sample Type	Hypervariable regions	DNA extraction method	Country of origin	Sequencing platform	Accession number for BioProject	Studies
**Diarrhea or health piglet**	Health piglet	Yes	12	12			feces	V4	TIANamp Stool DNA Kit	China	Illumina HiSeq 2500	PRJNA340296	(17)
**Diarrhea or health piglet**	Health piglet	NA	24				feces	V3-V4	TIANGEN DNA extract kit	China	Illumina MiSeq	PRJNA450515	(18)
**Bacilllus or antibiotics**	NC	No	18	36			feces	V1-V3	E.Z.N. A. stool DNA Kit	Denmark	Illumina MiSeq	PRJNA503676	(3)
**Birth weight**	Normal birth weight	Yes	18	12			feces	V3-V4	QIAamp Fast DNA Stool Mini Kit	China	Illumina HiSeq 2500	PRJNA448808	(2)
**Age**	Differe nt age	NA	17	51			feces	V3-V4	Godon 1997	China	Illumina Miseq	PRJNA381010	(4)
**Age**	Differe nt age	NA		20			feces	V5-V6	QIAamp Fast DNA Stool Mini Kit	South Korea	Illumina Hi-Seq 2000	PRJNA437010	(5)
**Age**	Differe nt age	Yes	31	93	31		feces	V3-V4	Lepage et al., 2005	France	454 FLX	PRJNA266269	(19)
**Antibiotic and feed additive**	NC	NA		15			feces	V1-V2	Power Faecal™DNA isolation kit	Spain	Ion Torrent PGM	PRJNA445806	(7)

**Body weight**	High body weight	NA			18		feces	V4	NucleoSpin® soil kit	South Korea	Illumina MiSeq	PRJNA336436	(14)
**Antibiotic**	NC	NA			30	20	feces	V3	QIAgen DNA Mini Stool kit	USA	454 FLX		(16)
**Antibiotic**	NC	NA				7	feces	V3	Powermax soil isolation kit (MoBio)	USA	454 FLX	PRJNA59781	(20)
**Antibiotic**	NC	NA			11		feces	V1-V3	A sterile glass microscope slide method	USA	454 FLX	PRJNA72355	(21)
**Antibiotic**	NC	NA		42	30		feces	V1-V3	PowerBiome DNA Isolation Kit	USA	454 FLX	PRJNA237795	(10)
**Age**	Different age	NA	35	67	83	67	feces	V4	PowerLyzer PowerSoil DNA Isolation Kit	USA	Illumina Miseq	PRJNA531671	(22)
**Age**	Different age	Yes	32	32	64	32	feces	V4	NucleoSpin®Soil Kit	South Korea	Illumina Miseq	PRJNA336436	(14)
**Age**	Different age	NO	121	111			feces	V4	Zymo Research Fecal DNA kit	USA	Illumina Miseq	PRJEB20514	(23)

Table S4. The relative abundance of microbial phyla detected in the gut of pigs at each growth stage based on the collected sequences

Phyla	Lactation, %	Nursery, %	Growing, %	Finishing, %
Proteobacteria	17.3543	4.3482	3.3167	4.0274
Firmicutes	43.7504	52.2323	50.2444	54.5900
Bacteroidetes	29.1791	37.0922	39.5374	32.8301
Tenericutes	0.0738	1.0031	0.4363	0.7101
Fusobacteria	5.4459	0.8923	0.0009	0.5826
Spirochaetes	0.4599	1.2865	3.2155	2.2741
Actinobacteria	1.5073	1.4474	1.2095	1.4326
Verrucomicrobia	0.4955	0.1253	0.0211	0.5639
Synergistetes	0.6601	0.2428	0.0115	0.0284
Deferribacteres	0.0013	0.0549	0.0103	0.0022
OP9	0.0000	0.0185	0.0000	0.0000
WPS-2	0.0238	0.0100	0.1917	0.1651
Chloroflexi	0.0000	0.0191	0.0000	0.0000
Cyanobacteria	0.0337	0.2203	0.3009	0.1987
Lentisphaerae	0.0294	0.0537	0.0849	0.0482
TM7	0.0018	0.0492	0.1082	0.0023
Planctomycetes	0.0518	0.0486	0.1626	0.1794
OP8	0.0000	0.0079	0.0000	0.0000
Elusimicrobia	0.0097	0.0229	0.0228	0.0232
WWE1	0.0000	0.0048	0.0000	0.0000
Fibrobacteres	0.0027	0.0328	0.0503	0.0456
Chlorobi	0.0000	0.0034	0.0000	0.0000
Thermi	0.0035	0.0033	0.0000	0.0000
Acidobacteria	0.0004	0.0026	0.0000	0.0000
Gemmatimonadetes	0.0001	0.0027	0.0000	0.0000
Chlamydiae	0.0000	0.0026	0.0054	0.0029
WS3	0.0000	0.0006	0.0000	0.0000
Hyd24-12	0.0000	0.0004	0.0000	0.0000
OP3	0.0000	0.0004	0.0000	0.0000
GN02	0.0000	0.0003	0.0000	0.0001
WS1	0.0000	0.0003	0.0000	0.0000
Armatimonadetes	0.0000	0.0003	0.0000	0.0000
GN04	0.0000	0.0003	0.0000	0.0000
NKB19	0.0000	0.0001	0.0000	0.0001
BRC1	0.0000	0.0001	0.0000	0.0000
SAR406	0.0000	0.0001	0.0000	0.0000
WS5	0.0000	0.0000	0.0000	0.0000
OD1	0.0000	0.0000	0.0000	0.0000
Caldithrix	0.0000	0.0001	0.0000	0.0000
WS2	0.0000	0.0000	0.0000	0.0000
OP11	0.0000	0.0000	0.0000	0.0000

AC1	0.0000	0.0000	0.0000	0.0000
Nitrospirae	0.0000	0.0000	0.0000	0.0000
TM6	0.0000	0.0001	0.0000	0.0000
Others	0.9156	0.7693	1.0697	2.2931

Table S5. The relative abundance of microbial genera in the gut of pigs at each growth stage based on the collected sequences

Genus	Lactation, %	Nursery, %	Growing, %	Finishing, %
Escherichia	12.4481	1.1865	0.0555	0.5888
Lactobacillus	8.6713	6.2465	3.0027	4.4041
Prevotellaceae_Prevotella	5.7273	22.3193	27.7902	15.2487
Clostridiaceae_Clostridium	2.6426	0.6363	0.1051	3.0647
Bacteroides	15.5925	2.2629	0.0640	0.5838
Oscillospira	2.5190	2.8341	1.2773	1.7942
Fusobacterium	4.2181	0.7756	0.0006	0.5399
Paraprevotellaceae_Prevotella	0.7694	2.0456	2.0939	3.2970
Treponema	0.3506	1.1544	2.9578	2.0853
Bacillaceae_Bacillus	0.4710	0.8919	0.0044	0.0017
Streptococcus	1.2605	1.2482	4.2759	5.8591
Veillonella	0.6344	0.0566	0.0014	0.0075
Megasphaera	0.6619	2.3505	5.3453	2.6752
Phascolarctobacterium	1.1993	1.7962	1.3155	1.8754
Flexispira	0.1743	0.2863	0.0165	0.0107
Lachnospiraceae_Ruminococcus	2.7682	0.7492	0.2268	0.1009
Odoribacter	0.2869	0.0225	0.0015	0.0005
Trabulsiella	0.0011	0.1292	0.0002	0.0000
Blautia	0.2056	2.3868	2.1037	0.7764
Actinobacillus	0.4023	0.0442	0.0040	0.0058
Roseburia	0.3129	1.7117	1.1677	0.5067
Parabacteroides	1.1895	1.0870	0.6371	0.9023
Akkermansia	0.4751	0.0951	0.0010	0.2508
p-75-a5	0.4036	0.8009	0.0722	0.2187
Ruminococcaceae_Ruminococcus	1.9182	1.4709	1.2421	1.1973
Synergistes	0.5589	0.0732	0.0006	0.0015
Campylobacter	0.2300	0.4045	0.2869	0.3636
Catenibacterium	0.0243	0.6437	0.3391	0.0834
Eubacterium	1.3447	0.9964	0.4138	0.1706
Collinsella	0.3077	0.5806	0.3956	0.2859
CF231	0.0680	0.8227	0.5012	1.2401
Ralstonia	0.0048	0.0023	0.0000	0.1625
Anaerovibrio	0.3003	0.8526	1.0438	1.4554
02d06	0.1233	0.4517	0.3179	0.2423
Comamonas	0.1007	0.0003	0.0000	0.0001
Coprococcus	0.0776	0.7907	0.8687	0.6356
SMB53	0.1187	0.3270	0.9424	2.1544
Succinivibrio	0.0142	0.5707	1.0078	0.5982
Succiniclasticum	0.0609	0.0008	0.0024	0.0000
Pyramidobacter	0.0771	0.1367	0.0000	0.0001
Actinomyces	0.1370	0.0062	0.0008	0.1036

Genus	Lactation, %	Nursery, %	Growing, %	Finishing, %
Lachnospiraceae_Clostridium	1.1702	0.1563	0.0484	0.0184
YRC22	0.0114	0.1744	0.3066	2.3919
Faecalibacterium	0.1524	2.1900	2.2010	0.5909
Dorea	0.7373	0.7271	0.4923	0.2939
Mitsuokella	0.1300	0.3504	0.3640	0.2005
Pasteurella	0.1600	0.0026	0.0000	0.0000
Trueperella	0.1953	0.0110	0.0006	0.0004
Mucispirillum	0.0013	0.0549	0.0103	0.0022
Bifidobacterium	0.4380	0.3469	0.2514	0.2317
Peptostreptococcus	0.2173	0.0073	0.0004	0.0324
Turicibacter	0.1416	0.0817	0.2430	1.1301
Erysipelotrichaceae_Clostridium	0.1445	0.0344	0.0001	0.0008
Butyricimonas	0.5904	0.0791	0.0019	0.0019
Megamonas	0.0056	0.0400	0.0000	0.0003
Gallibacterium	0.0245	0.0214	0.0000	0.0000
Butyricicoccus	0.1025	0.3871	0.4439	0.1296
Sharpea	0.0283	0.1351	0.2302	0.0469
Yersinia	0.0007	0.0003	0.0000	0.0746
Acidaminococcus	0.1172	0.2992	0.5814	0.0721
Proteus	0.0791	0.0012	0.0000	0.0002
Sphaerochaeta	0.1092	0.1256	0.2550	0.1720
RFN20	0.0151	0.0546	0.0723	0.0328
Anaerococcus	0.0734	0.0253	0.0312	0.1682
Staphylococcus	0.2045	0.0406	0.0006	0.0031
Dialister	0.0867	0.2413	0.6658	0.2758
Sutterella	0.2163	0.1285	0.1354	0.0720
Desulfovibrio	0.5700	0.2672	0.1432	0.2086
Enterococcus	0.0733	0.0213	0.0000	0.0007
Parvimonas	0.0186	0.0139	0.0005	0.0406
Mogibacterium	0.1863	0.1374	0.0988	0.1420
Bulleidia	0.0802	0.2544	0.3031	0.1484
Desulfococcus	0.0000	0.0100	0.0000	0.0000
Shuttleworthia	0.0001	0.0115	0.0973	0.0364
Butyrivibrio	0.0006	0.0181	0.1437	0.0888
T78	0.0000	0.0095	0.0000	0.0000
Paludibacter	0.0146	0.0138	0.0112	0.1949
Anaerobiospirillum	0.0337	0.0137	0.0043	0.0005
Morganella	0.0514	0.0009	0.0000	0.0000
Peptoniphilus	0.0770	0.0155	0.0116	0.0789
ph2	0.0329	0.0122	0.0003	0.0031
Aerococcus	0.0100	0.0016	0.0026	0.0330
Porphyromonas	0.0368	0.0123	0.0000	0.0159
1-68	0.0006	0.0006	0.0059	0.0542

Genus	Lactation, %	Nursery, %	Growing, %	Finishing, %
Bilophila	0.0927	0.0374	0.0002	0.0003
Sphingobium	0.0094	0.0000	0.0000	0.0000
Finegoldia	0.0348	0.0054	0.0049	0.0469
Helcococcus	0.0474	0.0035	0.0000	0.0002
Lachnospira	0.0072	0.1497	0.1275	0.0626
Pseudoramibacter_Eubacterium	0.0154	0.0373	0.0357	0.0017
Acinetobacter	0.0524	0.0126	0.0000	0.0062
Moraxella	0.0694	0.0050	0.0010	0.0010
Delftia	0.0059	0.0029	0.0000	0.0171
Candidatus Cloacamonas	0.0000	0.0048	0.0000	0.0000
Peptococcus	0.0953	0.0601	0.0671	0.1890
Fibrobacter	0.0027	0.0325	0.0503	0.0456
Allobaculum	0.0002	0.0128	0.0011	0.0040
Dinoroseobacter	0.0000	0.0036	0.0000	0.0000
Ruminobacter	0.0001	0.0081	0.0000	0.0032
Oribacterium	0.0116	0.0946	0.2063	0.1344
Mobiluncus	0.0004	0.0003	0.0019	0.0292
Anaerofilum	0.0083	0.0073	0.0005	0.0003
Corynebacterium	0.0477	0.0198	0.0021	0.0141
Selenomonas	0.0017	0.0159	0.0089	0.0234
Sarcina	0.0007	0.0011	0.0038	0.0562
Lachnobacterium	0.0000	0.0098	0.0000	0.0000
B-42	0.0000	0.0028	0.0000	0.0000
Arcanobacterium	0.0171	0.0004	0.0017	0.0227
Tissierella_Soehngenia	0.0001	0.0026	0.0000	0.0000
Psychrobacter	0.0038	0.0007	0.0004	0.0094
Lactococcus	0.0058	0.0018	0.0002	0.0020
Anaerotruncus	0.0294	0.0081	0.0002	0.0001
Trichococcus	0.0044	0.0003	0.0000	0.0014
Eggerthella	0.0510	0.0025	0.0000	0.0001
Cloacibacillus	0.0042	0.0009	0.0000	0.0000
Aggregatibacter	0.0102	0.0005	0.0005	0.0002
Coprobacillus	0.0012	0.0038	0.0379	0.0046
Anaerostipes	0.0003	0.0104	0.0317	0.0153
Thiobacillus	0.0000	0.0017	0.0000	0.0000
Wautersiella	0.0029	0.0000	0.0000	0.0021
Anaeroplasma	0.0008	0.0054	0.0040	0.0052
Atopobium	0.0060	0.0001	0.0000	0.0002
Desulfocapsa	0.0000	0.0015	0.0000	0.0000
Facklamia	0.0134	0.0018	0.0001	0.0035
Deinococcus	0.0027	0.0000	0.0000	0.0000
L7A_E11	0.0081	0.0272	0.0392	0.0382
Asteroleplasma	0.0004	0.0057	0.0079	0.0045

Genus	Lactation, %	Nursery, %	Growing, %	Finishing, %
Jeotgalicoccus	0.0031	0.0002	0.0000	0.0009
Pseudomonas	0.0249	0.0099	0.0000	0.0012
Shewanella	0.0021	0.0008	0.0000	0.0094
Devosia	0.0001	0.0013	0.0000	0.0000
Peptostreptococcaceae_Clostridium	0.0055	0.0000	0.0000	0.0001
Haemophilus	0.0189	0.0018	0.0000	0.0047
Epulopiscium	0.0086	0.0020	0.0021	0.0811
Victivallis	0.0038	0.0013	0.0012	0.0002
Kocuria	0.0164	0.0013	0.0000	0.0007
Christensenella	0.0061	0.0056	0.0000	0.0000
Desulfotignum	0.0000	0.0010	0.0000	0.0000
Desulfobulbus	0.0000	0.0009	0.0000	0.0000
Holdemania	0.0192	0.0019	0.0000	0.0000
Thiocapsa	0.0000	0.0008	0.0000	0.0000
Adlercreutzia	0.0021	0.0012	0.0001	0.0062
Oxalobacter	0.0028	0.0108	0.0300	0.0105
rc4-4	0.0026	0.0119	0.0063	0.0223
Slackia	0.0020	0.0103	0.0424	0.0604
Chlamydia	0.0000	0.0026	0.0054	0.0028
Solibacillus	0.0017	0.0001	0.0000	0.0000
Desulfosarcina	0.0000	0.0006	0.0000	0.0000
Elizabethkingia	0.0010	0.0000	0.0000	0.0000
Pyropia	0.0000	0.0000	0.0000	0.0016
Sphingobacterium	0.0014	0.0000	0.0000	0.0000
gut	0.0013	0.0017	0.0002	0.0011
Schwartzia	0.0000	0.0027	0.0014	0.0024
Myroides	0.0012	0.0000	0.0000	0.0000
Anaerovorax	0.0000	0.0032	0.0000	0.0013
Ignavibacterium	0.0000	0.0005	0.0000	0.0000
Arthrobacter	0.0012	0.0001	0.0000	0.0004
Edwardsiella	0.0000	0.0000	0.0000	0.0043
Ruminococcaceae_Clostridium	0.0024	0.0004	0.0015	0.0055
Maribacter	0.0000	0.0000	0.0000	0.0011
SHD-231	0.0000	0.0004	0.0000	0.0000
Rothia	0.0053	0.0022	0.0000	0.0000
Paraprevotella	0.0000	0.0007	0.0001	0.0001
Providencia	0.0006	0.0000	0.0000	0.0000
Macrococcus	0.0021	0.0002	0.0001	0.0026
Micrococcus	0.0011	0.0002	0.0000	0.0004
Anaerofustis	0.0007	0.0026	0.0013	0.0029
Rhodobacter	0.0004	0.0004	0.0000	0.0000
Plesiocystis	0.0000	0.0003	0.0000	0.0000

Genus	Lactation, %	Nursery, %	Growing, %	Finishing, %
Rhodococcus	0.0002	0.0003	0.0000	0.0000
Tindallia_Anoxynatronum	0.0000	0.0003	0.0000	0.0000
Elusimicrobium	0.0020	0.0007	0.0000	0.0005
Vagococcus	0.0056	0.0008	0.0000	0.0003
Pelobacter	0.0000	0.0003	0.0000	0.0000
Robinsoniella	0.0055	0.0011	0.0000	0.0000
Gemella	0.0007	0.0006	0.0000	0.0000
Helicobacter	0.0000	0.0010	0.0000	0.0000
Dehalobacterium	0.0029	0.0072	0.0088	0.0103
Flavobacterium	0.0000	0.0004	0.0000	0.0000
KSA1	0.0000	0.0004	0.0000	0.0000
Fusibacter	0.0000	0.0003	0.0000	0.0000
Gillisia	0.0000	0.0003	0.0000	0.0000
Alistipes	0.0009	0.0004	0.0000	0.0000
Fontibacter	0.0000	0.0003	0.0000	0.0000
Mycoplasma	0.0000	0.0025	0.0008	0.0005
Pseudobutyrivibrio	0.0003	0.0008	0.0079	0.0177
HTCC	0.0000	0.0011	0.0000	0.0000
Weissella	0.0005	0.0009	0.0000	0.0000
Ureaplasma	0.0000	0.0005	0.0000	0.0000
Anoxybacillus	0.0002	0.0003	0.0001	0.0001
Acidovorax	0.0003	0.0000	0.0000	0.0000
Proteiniclasticum	0.0004	0.0000	0.0000	0.0000
Planococcaceae_Bacillus	0.0087	0.0034	0.0000	0.0000
Spirochaeta	0.0000	0.0003	0.0000	0.0000
Glaciecola	0.0000	0.0000	0.0000	0.0005
Hydrogenophaga	0.0003	0.0002	0.0000	0.0000
Mycoplana	0.0032	0.0015	0.0000	0.0000
Flexithrix	0.0000	0.0000	0.0000	0.0004
Chryseobacterium	0.0006	0.0002	0.0000	0.0001
Allofustis	0.0000	0.0002	0.0000	0.0000
Halothiobacillus	0.0000	0.0002	0.0000	0.0000
Dysgonomonas	0.0003	0.0000	0.0000	0.0000
C1_B004	0.0000	0.0001	0.0000	0.0000
Enhydrobacter	0.0015	0.0005	0.0002	0.0004
Salegentibacter	0.0000	0.0001	0.0000	0.0000
Granulicatella	0.0014	0.0003	0.0000	0.0002
Novosphingobium	0.0003	0.0000	0.0000	0.0000
Stenotrophomonas	0.0006	0.0004	0.0000	0.0001
Agrobacterium	0.0030	0.0010	0.0000	0.0000
Brachybacterium	0.0013	0.0003	0.0000	0.0002
Leuconostoc	0.0005	0.0008	0.0000	0.0003
Thioalkalivibrio	0.0000	0.0001	0.0000	0.0000

Genus	Lactation, %	Nursery, %	Growing, %	Finishing, %
Arthrospira	0.0003	0.0001	0.0000	0.0000
Cloacibacterium	0.0010	0.0004	0.0000	0.0000
Erysipelothrix	0.0003	0.0000	0.0000	0.0000
Leptotrichia	0.0008	0.0003	0.0000	0.0000
Bradyrhizobium	0.0001	0.0002	0.0000	0.0007
Zymomonas	0.0000	0.0001	0.0000	0.0000
Carnobacterium	0.0002	0.0000	0.0000	0.0000
Phaeobacter	0.0000	0.0000	0.0000	0.0003
Wohlfahrtiimonas	0.0002	0.0001	0.0000	0.0000
Dietzia	0.0004	0.0003	0.0001	0.0001
Abiotrophia	0.0002	0.0002	0.0000	0.0000
Sphingomonas	0.0019	0.0014	0.0000	0.0001
Desulfosalsimonas	0.0000	0.0001	0.0000	0.0000
Rheinheimera	0.0019	0.0010	0.0000	0.0000
wall-less	0.0000	0.0002	0.0000	0.0000
Blastococcus	0.0010	0.0006	0.0000	0.0000
Gemmatimonas	0.0000	0.0001	0.0000	0.0000
Paracoccus	0.0013	0.0007	0.0000	0.0001
Pantoea	0.0000	0.0006	0.0000	0.0000
Alkanindiges	0.0011	0.0006	0.0000	0.0000
Arcobacter	0.0001	0.0001	0.0001	0.0002
Brevibacterium	0.0003	0.0000	0.0000	0.0001
Acidaminobacter	0.0000	0.0001	0.0000	0.0000
Cellulosimicrobium	0.0001	0.0001	0.0000	0.0001
Alcaligenes	0.0001	0.0000	0.0000	0.0000
Alicyclobacillus	0.0003	0.0003	0.0000	0.0000
Desulfonema	0.0000	0.0001	0.0000	0.0000
Blvii28	0.0000	0.0005	0.0000	0.0000
Rhodanobacter	0.0000	0.0002	0.0000	0.0000
Virgibacillus	0.0010	0.0004	0.0000	0.0001
Phenylobacterium	0.0009	0.0002	0.0000	0.0000
Leucobacter	0.0002	0.0003	0.0000	0.0003
Thermus	0.0006	0.0003	0.0000	0.0000
Neisseria	0.0007	0.0001	0.0000	0.0000
Candidatus Arthromitus	0.0000	0.0001	0.0000	0.0000
Dethiosulfatibacter	0.0000	0.0001	0.0000	0.0000
Roseomonas	0.0001	0.0000	0.0000	0.0000
Buchnera	0.0000	0.0001	0.0000	0.0000
Fructobacillus	0.0000	0.0001	0.0000	0.0000
PSB-M-3	0.0000	0.0001	0.0000	0.0000
Caldinitratiruptor	0.0005	0.0003	0.0000	0.0000
Rubellimicrobium	0.0001	0.0001	0.0000	0.0000
5-7N15	0.0001	0.0004	0.0000	0.0000

Genus	Lactation, %	Nursery, %	Growing, %	Finishing, %
Leucothrix	0.0000	0.0000	0.0000	0.0001
Luteolibacter	0.0000	0.0001	0.0000	0.0000
Loktanella	0.0000	0.0005	0.0000	0.0000
Aquiflexum	0.0000	0.0003	0.0000	0.0000
Alloiococcus	0.0001	0.0000	0.0000	0.0000
Gallicola	0.0001	0.0000	0.0002	0.0002
Marinobacter	0.0000	0.0000	0.0000	0.0000
Cupriavidus	0.0007	0.0003	0.0000	0.0000
Desemzia	0.0001	0.0000	0.0000	0.0002
Geobacillus	0.0001	0.0001	0.0000	0.0000
Pleomorphomonas	0.0004	0.0004	0.0000	0.0000
Lysobacter	0.0001	0.0001	0.0000	0.0000
Syntrophomonas	0.0000	0.0002	0.0006	0.0006
Aeromicrobium	0.0001	0.0002	0.0000	0.0000
Kaistobacter	0.0002	0.0001	0.0000	0.0000
Methylobacterium	0.0000	0.0001	0.0000	0.0000
Sulfurimonas	0.0000	0.0000	0.0000	0.0000
Meiothermus	0.0002	0.0002	0.0000	0.0000
ecb11	0.0000	0.0001	0.0000	0.0000
Telmatospirillum	0.0000	0.0000	0.0000	0.0000
Pseudoalteromonadaceae_Vibrio	0.0002	0.0000	0.0000	0.0000
Mycobacterium	0.0000	0.0002	0.0001	0.0001
Propionibacterium	0.0001	0.0000	0.0000	0.0002
Tessaracoccus	0.0001	0.0000	0.0000	0.0000
Aquicella	0.0000	0.0001	0.0000	0.0000
Desulfobacter	0.0000	0.0000	0.0000	0.0000
Ochrobactrum	0.0001	0.0001	0.0001	0.0001
Oceanobacillus	0.0000	0.0000	0.0003	0.0004
Sporosarcina	0.0000	0.0000	0.0001	0.0000
Planctomyces	0.0000	0.0000	0.0000	0.0000
Pediococcus	0.0001	0.0000	0.0000	0.0000
Dermabacter	0.0000	0.0001	0.0000	0.0000
Nesterenkonia	0.0001	0.0000	0.0000	0.0000
Rhodopirellula	0.0000	0.0000	0.0000	0.0001
Bdellovibrio	0.0000	0.0002	0.0000	0.0000
Ignatzschineria	0.0000	0.0001	0.0001	0.0000
Hydrogenophilus	0.0000	0.0000	0.0000	0.0001
Salinibacter	0.0000	0.0003	0.0000	0.0000
Streptomyces	0.0000	0.0000	0.0002	0.0000
Renibacterium	0.0000	0.0000	0.0000	0.0000
Novispirillum	0.0000	0.0000	0.0000	0.0000
Methylotenera	0.0000	0.0000	0.0000	0.0001
Rhodoplanes	0.0000	0.0000	0.0000	0.0000

Genus	Lactation, %	Nursery, %	Growing, %	Finishing, %
WCHB1-05	0.0000	0.0000	0.0000	0.0000
Paenibacillus	0.0000	0.0000	0.0003	0.0000
Guggenheimella	0.0000	0.0001	0.0000	0.0000
Psychroserpens	0.0000	0.0000	0.0000	0.0001
BD2-6	0.0000	0.0000	0.0000	0.0000
Filifactor	0.0000	0.0000	0.0000	0.0000
Fluviicola	0.0001	0.0000	0.0000	0.0000
Alkaliphilus	0.0000	0.0000	0.0000	0.0000
Clostridiisalibacter	0.0000	0.0000	0.0000	0.0000
Exiguobacterium	0.0001	0.0001	0.0000	0.0000
Pythium	0.0000	0.0000	0.0000	0.0000
Pseudoxanthomonas	0.0000	0.0000	0.0000	0.0000
Eikenella	0.0000	0.0000	0.0000	0.0000
Cetobacterium	0.0001	0.0000	0.0000	0.0001
WH1-8	0.0000	0.0000	0.0000	0.0000
Methylophaga	0.0000	0.0000	0.0000	0.0000
Caldilinea	0.0000	0.0000	0.0000	0.0000
Saccharopolyspora	0.0000	0.0000	0.0001	0.0000
Coxiella	0.0000	0.0000	0.0000	0.0000
Alteromonas	0.0000	0.0000	0.0000	0.0000
Steroidobacter	0.0000	0.0000	0.0000	0.0000
Asticcacaulis	0.0000	0.0000	0.0000	0.0000
Vibrionaceae_Vibrio	0.0000	0.0000	0.0000	0.0000
Flavisolibacter	0.0000	0.0000	0.0000	0.0000
Acholeplasma	0.0000	0.0000	0.0000	0.0000
Syntrophobacter	0.0000	0.0000	0.0000	0.0000
Candidatus Azobacteroides	0.0000	0.0000	0.0000	0.0000
Geobacter	0.0000	0.0000	0.0000	0.0000
Rhodospirillum	0.0000	0.0000	0.0000	0.0000
Sporotomaculum	0.0000	0.0000	0.0000	0.0000
Others	23.9454	31.4913	32.2112	38.5129

Table S6. The relative abundance of annotate microbial species in the gut of pigs at each growth stage based on the collected sequences

Taxonomy	Lactation, %	Nursery, %	Growing, %	Finishing, %
Escherichia coli	12.4481	1.1865	0.0555	0.5888
Prevotella copri	0.1822	10.7031	13.3712	4.8308
Clostridium perfringens	1.8906	0.1149	0.0004	0.0471
Bacteroides fragilis	5.9585	0.6790	0.0003	0.0306
Bacillus coagulans	0.4647	0.8895	0.0000	0.0000
Flexispira rappini	0.1696	0.2629	0.0104	0.0106
Ruminococcus gnavus	2.5461	0.5781	0.1861	0.0938
Prevotella stercorea	2.5637	2.7214	0.7993	0.6271
Lactobacillus delbrueckii	0.8107	0.0617	0.0007	0.0000
Streptococcus luteciae	0.3339	0.8375	3.8490	3.4286
Bacteroides uniformis	0.3134	0.0467	0.0002	0.0008
Akkermansia muciniphila	0.4746	0.0873	0.0010	0.0031
Roseburia faecis	0.0996	0.1766	0.0395	0.0000
Bacteroides plebeius	0.3429	0.0588	0.0002	0.1428
Lactobacillus reuteri	1.6345	0.7793	0.1290	0.7923
Eubacterium dolichum	0.8142	0.0488	0.0000	0.0004
Parabacteroides distasonis	0.7165	0.2999	0.0001	0.0010
Lactobacillus mucosae	0.8684	0.3292	0.1625	0.0175
Eubacterium biforme	0.2801	0.7100	0.3202	0.1074
Pyramidobacter piscolens	0.0771	0.1366	0.0000	0.0001
Clostridium citroniae	0.9022	0.0873	0.0000	0.0003
Faecalibacterium prausnitzii	0.0881	1.8271	1.8674	0.4587
Bacteroides caccae	0.2738	0.0948	0.0000	0.0003
Bacteroides coprophilus	0.1755	0.0448	0.0019	0.0098
Blautia obeum	0.0167	0.3641	0.2836	0.0558
Blautia producta	0.0477	0.0364	0.3123	0.0008
Pasteurella aerogenes	0.1600	0.0026	0.0000	0.0000
Bacteroides ovatus	0.1424	0.0207	0.0000	0.0000
Mucispirillum schaedleri	0.0013	0.0549	0.0103	0.0022
Collinsells aerofaciens	0.1640	0.2631	0.1954	0.1338
Actinobacillus porcinus	0.1140	0.0183	0.0001	0.0004
Lactobacillus vaginalis	0.1791	0.0276	0.0000	0.0000
Gallibacterium genomosp.	0.0245	0.0214	0.0000	0.0000
Mitsuokella multacida	0.0248	0.2067	0.1572	0.0445
Ruminococcus torques	0.1346	0.0136	0.0001	0.0000
Butyricicoccus pullicaecorum	0.1025	0.3871	0.4439	0.1296
Sharpea azabuensis	0.0267	0.1222	0.0157	0.0235
Streptoccus alactolyticus	0.0008	0.0881	0.0038	0.0122
Coprococcus catus	0.0106	0.1672	0.1003	0.1163
Clostridium butyricum	0.0309	0.0637	0.0275	1.3636

Taxonomy	Lactation, %	Nursery, %	Growing, %	Finishing, %
Ruminococcus flavefaciens	0.0028	0.0672	0.0186	0.3374
Staphylococcus sciuri	0.0001	0.0122	0.0000	0.0000
Lactobacillus ruminis	0.0876	0.0735	0.1275	0.1354
Enterococcus cecorum	0.0682	0.0187	0.0000	0.0007
Shrpea p-3329-23G2	0.0016	0.0129	0.2144	0.0234
Clostridium hathewayi	0.2675	0.0661	0.0316	0.0144
Bulleidia moorei	0.0472	0.0319	0.0001	0.0016
Peptostreptococcus anaerobius	0.1168	0.0071	0.0004	0.0324
Lactobacillus salivarius	0.0172	0.0440	0.0000	0.0000
Bifidobacterium adolescentis	0.0423	0.0285	0.0475	0.0573
Ruminococcus bromii	0.0090	0.1108	0.1596	0.1256
Bulleidia p-1630-c5	0.0088	0.1712	0.2582	0.1356
Porphyromonas endodontalis	0.0047	0.0102	0.0000	0.0000
Bacteroides acidifaciens	0.0220	0.0005	0.0000	0.0000
Dorea formicigenerans	0.0076	0.1353	0.0942	0.0210
Actinomyces hyovaginalis	0.0224	0.0007	0.0000	0.0001
Veillonella dispar	0.0929	0.0084	0.0001	0.0039
Eubacterium cylindroides	0.0008	0.0191	0.0611	0.0091
Coprococcus eutactus	0.0045	0.0350	0.0709	0.0250
Fibrobacter succinogenes	0.0004	0.0087	0.0000	0.0119
Acinetobacter lwoffii	0.0129	0.0034	0.0000	0.0060
Bacillus cereus	0.0057	0.0013	0.0000	0.0000
Clostridium celatum	0.0034	0.0011	0.0000	0.1453
Streptoccus minor	0.0167	0.0030	0.0000	0.0000
Selenomonas ruminantium	0.0017	0.0159	0.0030	0.0207
Bacteroides eggerthii	0.0023	0.0062	0.0000	0.0002
Staphylococcus aureus	0.0032	0.0024	0.0003	0.0006
Lactobacillus coleohominis	0.0235	0.0196	0.0002	0.0000
Eggerthella lenta	0.0510	0.0025	0.0000	0.0001
Ruminococcus callidus	0.0010	0.0185	0.0272	0.0047
Desulfovibrio D168	0.0001	0.0028	0.0392	0.0983
Corynebacterium stationis	0.0031	0.0022	0.0005	0.0035
Actinobacillus seminis	0.0032	0.0002	0.0001	0.0004
Asteroleplasma anaerobium	0.0004	0.0057	0.0079	0.0045
Shewanella algae	0.0011	0.0005	0.0000	0.0094
Jeotgalicoccus psychrophilus	0.0019	0.0001	0.0000	0.0000
Clostridium difficile	0.0055	0.0000	0.0000	0.0000
Clostridium colinum	0.0000	0.0004	0.0140	0.0004
Lactobacilllus zeae	0.0269	0.0147	0.0000	0.0002
Lactobacillus garvieae	0.0018	0.0000	0.0000	0.0000
Haemophilus parasuis	0.0189	0.0018	0.0000	0.0047

Taxonomy	Lactation, %	Nursery, %	Growing, %	Finishing, %
Victivalllis vadensis	0.0038	0.0013	0.0012	0.0002
Sphingobium xenophagum	0.0016	0.0000	0.0000	0.0000
Pseudomonas viridiflava	0.0054	0.0009	0.0000	0.0000
Corynebacterium variabile	0.0015	0.0030	0.0002	0.0020
Pseudomonas pseudoalcaligenes	0.0105	0.0054	0.0000	0.0000
Oxalobacter formigenes	0.0028	0.0108	0.0300	0.0105
Acinetobacter guillouiae	0.0013	0.0000	0.0000	0.0000
Bifidobacterium bifidum	0.0043	0.0006	0.0001	0.0000
Pyropia yezoensis	0.0000	0.0000	0.0000	0.0016
Gut metagenome	0.0013	0.0017	0.0002	0.0011
Desulfovibrio vulgaris	0.0019	0.0002	0.0000	0.0000
Psychrobacter pulmonis	0.0000	0.0004	0.0000	0.0016
Sphingobacterium mizutaii	0.0012	0.0000	0.0000	0.0000
Bifidobacterium longum	0.0012	0.0002	0.0000	0.0000
Lactobacillus agilis	0.0021	0.0034	0.0000	0.0000
Clostridium methylpentosum	0.0024	0.0004	0.0015	0.0054
Rothia mucilaginosa	0.0039	0.0016	0.0000	0.0000
Macrococcus caseolyticus	0.0021	0.0002	0.0001	0.0026
Rothia nasimurium	0.0005	0.0001	0.0000	0.0000
Robinsoniella peoriensis	0.0055	0.0011	0.0000	0.0000
Helicobacter apodemus	0.0000	0.0010	0.0000	0.0000
Pseudomonas stutzeri	0.0014	0.0007	0.0000	0.0009
Myroides odoratimimus	0.0007	0.0000	0.0000	0.0000
Alistipes indistinctus	0.0009	0.0004	0.0000	0.0000
Fontibacter flavus	0.0000	0.0003	0.0000	0.0000
Anoxybacillus kestanbolensis	0.0002	0.0003	0.0001	0.0001
Clostridium aminophilum	0.0001	0.0004	0.0000	0.0014
Bacillus thermoalkalophilus	0.0087	0.0034	0.0000	0.0000
Glaciecola polaris	0.0000	0.0000	0.0000	0.0005
Clostridium neonatale	0.0000	0.0011	0.0000	0.0000
Streptococcus agalactiae	0.0003	0.0000	0.0000	0.0000
Pseudomonas balearica	0.0046	0.0022	0.0000	0.0000
Weissella paramesenteroides	0.0000	0.0003	0.0000	0.0000
Allofustis seminis	0.0000	0.0002	0.0000	0.0000
Halothiobacillus hydrothermalis	0.0000	0.0002	0.0000	0.0000
Flavobacterium frigidarium	0.0000	0.0001	0.0000	0.0000
Chlamydia pecorum	0.0000	0.0001	0.0000	0.0000
Prevotella ruminicola	0.0000	0.0008	0.0003	0.0000
Arthrospira platensis	0.0003	0.0001	0.0000	0.0000
Pseudomonas fragi	0.0002	0.0002	0.0000	0.0001

Taxonomy	Lactation, %	Nursery, %	Growing, %	Finishing, %
Bifidobacterium pseudolongum	0.0005	0.0001	0.0001	0.0001
Staphylococcus equorum	0.0002	0.0000	0.0000	0.0000
Parabacteroides gordonii	0.0000	0.0002	0.0000	0.0000
Acinetobacter rhizosphaerae	0.0033	0.0011	0.0000	0.0000
Desulfosalsimonas propionicica	0.0000	0.0001	0.0000	0.0000
Wall-less spirochete	0.0000	0.0002	0.0000	0.0000
Stenotrophomonas acidaminiphila	0.0002	0.0000	0.0000	0.0000
Blastococcus aggregatus	0.0010	0.0006	0.0000	0.0000
Pantoea agglomerans	0.0000	0.0006	0.0000	0.0000
Arcobacter cryaerophilus	0.0001	0.0001	0.0000	0.0001
Oscillospira guilliermondii	0.0000	0.0000	0.0002	0.0038
Alcaligenes faecalis	0.0001	0.0000	0.0000	0.0000
Clostridium bowmanii	0.0000	0.0001	0.0000	0.0000
Desulfonema limicola	0.0000	0.0001	0.0000	0.0000
Catenibacterium mitsuokai	0.0000	0.0001	0.0000	0.0000
Neisseria subflava	0.0005	0.0001	0.0000	0.0000
Prevotella melaninogenica	0.0002	0.0001	0.0000	0.0000
Roseomonas aerilata	0.0001	0.0000	0.0000	0.0000
Caldinitratiruptor microaerophilus	0.0005	0.0003	0.0000	0.0000
Aquiflexum balticum	0.0000	0.0003	0.0000	0.0000
Paracoccus marcusii	0.0004	0.0003	0.0000	0.0000
Desulfovibrio C21_c20	0.0000	0.0000	0.0002	0.0006
Ruminococcus albus	0.0000	0.0000	0.0001	0.0005
Weissella confusa	0.0000	0.0003	0.0000	0.0000
Lactobacillus brevis	0.0000	0.0002	0.0001	0.0000
Pseudomonas alcaligenes	0.0003	0.0002	0.0000	0.0000
Propionibacterium acnes	0.0001	0.0000	0.0000	0.0002
Rhodopirellula baltica	0.0000	0.0000	0.0000	0.0001
Desulfococcus oleovorans	0.0000	0.0000	0.0000	0.0000
Lactobacillus paralimentarius	0.0000	0.0000	0.0000	0.0000
Alistipes massiliensis	0.0000	0.0000	0.0000	0.0000
Methylotenera mobilis	0.0000	0.0000	0.0000	0.0001
Roseomonas mucosa	0.0000	0.0000	0.0000	0.0000
Bacteroides barnesiae	0.0000	0.0000	0.0000	0.0000
Lactobacillus iners	0.0001	0.0000	0.0000	0.0000
Clostridium metallolevans	0.0000	0.0000	0.0000	0.0001
Megamonas hypermegale	0.0000	0.0000	0.0000	0.0000
Pythium ultimum	0.0000	0.0000	0.0000	0.0000

Taxonomy	Lactation, %	Nursery, %	Growing, %	Finishing, %
Bacteroides coprosuis	0.0001	0.0000	0.0000	0.0000
Sphingobacterium multivorum	0.0000	0.0000	0.0000	0.0000
Sphingobacterium faecium	0.0000	0.0000	0.0000	0.0000
Cetobacterium somerae	0.0001	0.0000	0.0000	0.0001
Clostridium thermopalmarium	0.0000	0.0000	0.0000	0.0000
Dysgonomonas gadei	0.0000	0.0000	0.0000	0.0000
Others	63.3033	74.3676	76.4438	85.6581

Table S7. Factors associated with the community structure of the swine gut microbiota as measured using weighted and unweighted UniFrac distances and Bray-Curtis and Binary-Jaccard dissimilarities

Parameter	Value											
	Unweighted UniFrac			Weighted UniFrac			Bray-Curtis			Binary-Jaccard		
	Pseudo-F ratio	R^2	*P* value	Pseudo-F ratio	R^2	*P* value	Pseudo-F ratio	R^2	*P* value	Pseudo-F ratio	R^2	*P* value
Age	6.026	0.248	0.001	10.540	0.365	0.001	6.054	0.249	0.001	5.050	0.216	0.001
Study	35.121	0.193	0.001	69.650	0.322	0.001	40.101	0.214	0.001	29.022	0.165	0.001
Weaning day	31.415	0.176	0.001	60.143	0.290	0.001	34.872	0.192	0.001	26.232	0.151	0.001
Creep feed	28.059	0.160	0.001	49.917	0.254	0.001	33.513	0.186	0.001	24.290	0.142	0.001
Growth stages	11.095	0.070	0.001	18.634	0.113	0.001	11.943	0.075	0.001	9.607	0.061	0.001
Enterotype	29.318	0.062	0.001	56.655	0.113	0.001	28.077	0.060	0.001	23.742	0.051	0.001
Origin	2.524	0.006	0.021	0.493	0.001	0.461	5.916	0.013	0.001	3.601	0.008	0.001
Platform	2.524	0.006	0.035	0.493	0.001	0.476	5.916	0.013	0.001	3.601	0.008	0.001

Reference

1. Wang J, Han Y, Meng F, Zhao J, Zhou Z, Fan H. 2017. Fecal microbiota succession of piglets from birth to post-weaning by 454 pyrosequencing analysis. Transactions of Tianjin University 23:211-220.
2. Li N, Huang S, Jiang L, Wang W, Li T, Zuo B, Li Z, Wang J. 2018. Differences in the gut microbiota establishment and metabolome characteristics between low- and normal-birth-weight piglets during early-life. Frontiers in Microbiology 9:1-16.
3. Poulsen AR, Jonge N, Nielsen JL, Hojberg O, Lauridsen C, Cutting SM, Canibe N. 2018. Impact of Bacillus spp. spores and gentamicin on the gastrointestinal microbiota of suckling and newly weaned piglets. PLoS One 13:e0207382.

4. Chen L, Xu Y, Chen X, Fang C, Zhao L, Chen F. 2017. The maturing development of gut microbiota in commercial piglets during the weaning transition. Frontiers in Microbiology 8:1-13.
5. Guevarra RB, Hong SH, Cho JH, Kim BR, Shin J, Lee JH, Kang BN, Kim YH, Wattanaphansak S, Isaacson RE, Song M, Kim HB. 2018. The dynamics of the piglet gut microbiome during the weaning transition in association with health and nutrition. J Anim Sci Biotechnol 9:54.
6. Li Y, Guo Y, Wen Z, Jiang X, Ma X, Han X. 2018. Weaning stress perturbs gut microbiome and its metabolic profile in piglets. Sci Rep 8:18068.
7. Soler C, Goossens T, Bermejo A, Migura-Garcia L, Cusco A, Francino O, Fraile L. 2018. Digestive microbiota is different in pigs receiving antimicrobials or a feed additive during the nursery period. PLoS ONE 13:e0197353.
8. Kaevska M, Lorencova A, Videnska P, Sedlar K, Provaznik I, Trckova M. 2016. Effect of sodium humate and zinc oxide used in prophylaxis of post-weaning diarrhoea on faecal microbiota composition in weaned piglets. Veterinární Medicína 61:328-336.
9. Pajarillo EAB, Chae JP, Balolong MP, Kim HB, Seo K-S, Kang D-K. 2014. Pyrosequencing-based analysis of fecal microbial communities in three purebred pig lines. Journal of Microbiology 52:646-651.
10. Looft T, Allen HK, Casey TA, Alt DP, Stanton TB. 2014. Carbadox has both temporary and lasting effects on the swine gut microbiota. Front Microbiol 5:276.
11. Mu C, Yang Y, Su Y, Zoetendal EG, Zhu W. 2017. Differences in microbiota membership along the gastrointestinal tract of piglets and their differential alterations following an early-life antibiotic intervention. Front Microbiol 8:797.
12. Zhang D, Ji H, Liu H, Wang S, Wang J, Wang Y. 2016. Changes in the diversity and composition of gut microbiota of weaned piglets after oral administration oflactobacillusor an antibiotic. Applied Microbiology and Biotechnology 100:10081-10093.
13. Yu T, Wang Y, Chen S, Hu M, Wang Z, Wu G, Ma X, Chen Z, Zheng C. 2017. Low-molecular-weight chitosan supplementation increases the population of Prevotella in the cecal contents of weanling pigs. Front Microbiol 8:2182.
14. Han GG, Lee JY, Jin GD, Park J, Choi YH, Chae BJ, Kim EB, Choi YJ. 2017. Evaluating the association between body weight and the intestinal microbiota of weaned piglets via 16S rRNA sequencing. Appl Microbiol Biotechnol 101:5903-5911.
15. Kim HB, Borewicz K, White BA, Singer RS, Sreevatsan S, Tu ZJ, Isaacson RE. 2011. Longitudinal investigation of the age-related bacterial diversity in the feces of commercial pigs. Veterinary Microbiology 153:124-133.
16. Kim HB, Borewicz K, White BA, Singer RS, Sreevatsan S, Tu ZJ, Isaacson RE. 2012. Microbial shifts in the swine distal gut in response to the treatment with antimicrobial growth promoter, tylosin. Proc Natl Acad Sci U S A 109:15485-15490.
17. Yang Q, Huang X, Zhao S, Sun W, Yan Z, Wang P, Li S, Huang W, Zhang S, Liu L, Gun S. 2017. Structure and function of the fecal microbiota in diarrheic neonatal piglets. Frontiers in Microbiology 8.

18. Han C, Dai Y, Liu B, Wang L, Wang J, Zhang J. 2019. Diversity analysis of intestinal microflora between healthy and diarrheal neonatal piglets from the same litter in different regions. Anaerobe 55:136-141.

19. Mach N, Berri M, Estellé J, Levenez F, Lemonnier G, Denis C, Leplat J-J, Chevaleyre C, Billon Y, Doré J, Rogel-Gaillard C, Lepage P. 2015. Early-life establishment of the swine gut microbiome and impact on host phenotypes. Environmental Microbiology Reports 7:554-569.

20. Looft T, Johnson TA, Allen HK, Bayles DO, Alt DP, Stedtfeld RD, Sul WJ, Stedtfeld TM, Chai B, Cole JR, Hashsham SA, Tiedje JM, Stanton TB. 2012. In-feed antibiotic effects on the swine intestinal microbiome. Proc Natl Acad Sci U S A 109:1691-1696.

21. Looft T, Allen HK, Cantarel BL, Levine UY, Bayles DO, Alt DP, Henrissat B, Stanton TB. 2014. Bacteria, phages and pigs: the effects of in-feed antibiotics on the microbiome at different gut locations. The ISME Journal 8:1566-1576.

22. Wang X, Tsai T, Deng F, Wei X, Chai J, Knapp J, Apple J, Maxwell CV, Lee JA, Li Y, Zhao J. 2019. Longitudinal investigation of the swine gut microbiome from birth to market reveals stage and growth performance associated bacteria. Microbiome 7:109-127.

23. Frese SA, Parker K, Calvert CC, Mills DA. 2015. Diet shapes the gut microbiome of pigs during nursing and weaning. Microbiome 3:28-38.
